# Community Compositions of Phytoplankton and Eukaryotes during the Mixing Periods of a Drinking Water Reservoir: Dynamics and Interactions

**DOI:** 10.3390/ijerph17041128

**Published:** 2020-02-11

**Authors:** Miaomiao Yan, Shengnan Chen, Tinglin Huang, Baoqin Li, Nan Li, Kaiwen Liu, Rongrong Zong, Yutian Miao, Xin Huang

**Affiliations:** 1 Shaanxi Key Laboratory of Environmental Engineering, Key Laboratory of Northwest Water Resource, Environment and Ecology, MOE, Xi’an University of Architecture and Technology, Xi’an 710055, China; Ymmzsj@163.com (M.Y.); huangtinglin@xauat.edu.cn (T.H.); kevin_wood1989@163.com (N.L.); lkw008866@163.com (K.L.); Rongrongzy_2019@163.com (R.Z.); miaoyutian728@163.com (Y.M.); xin_huang@xauat.edu.cn (X.H.); 2 Guangdong Key Laboratory of Integrated Agro-Environmental Pollution Control and Management, Guangdong Institute of Eco-Environmental Science and Technology, Guangzhou 510650, China; bqli@soil.gd.cn

**Keywords:** drinking water reservoir, phytoplankton, eukaryotes, coexistence and interaction, community composition, networks approach

## Abstract

In deep drinking water reservoir ecosystems, the dynamics and interactions of community compositions of phytoplankton and eukaryotes during the mixing periods are still unclear. Here, morphological characteristics combined with high-throughput DNA sequencing (HTS) were used to investigate the variations of phytoplankton and the eukaryotic community in a large canyon-shaped, stratified reservoir located at the Heihe River in Shaanxi Province for three months. The results showed that Bacillariophyta and Chlorophyta were the dominant taxa of the phytoplankton community, accounting for more than 97% of total phytoplankton abundance, which mainly consisted of *Melosira* sp., *Cyclotella* sp., and *Chlorella* sp., respectively. Illumina Miseq sequencing suggested that the biodiversity of eukaryotes increased over time and that species distribution was more even. Arthropoda (6.63% to 79.19%), Ochrophyta (5.60% to 35.16%), Ciliophora (1.81% to 10.93%) and Cryptomonadales (0.25% to 11.48%) were the keystone taxa in common, contributing over 50% of the total eukaryotic community. Cryptomycota as a unique fungus was observed to possess significant synchronization with algal density, reaching a maximum of 10.70% in December (when the algal density distinctly decreased) and suggesting that it might affect the growth of algae through parasitism. Co-occurrence network patterns revealed the complicated and diverse interactions between eukaryotes and phytoplankton, suggesting that eukaryotes respond to variations in dynamic structure of the phytoplankton community, although there might be antagonistic or mutualistic interactions between them. Redundancy analysis (RDA) results showed that environmental variables collectively explained a 96.7% variance of phytoplankton and 96.3% variance of eukaryotic microorganisms, indicating that the temporal variations of phytoplankton and eukaryotic microorganisms were significantly affected by environmental conditions. This study shows that potential interactions exist between phytoplankton and eukaryotic microorganism communities, andcould improve our understanding of the ecological roles of phytoplankton and eukaryotic microorganisms in changing aquatic ecosystems. However, long-term investigations are necessary in order to obtain comprehensive understandings of their complicated associations.

## 1. Introduction

Reservoirs have many important ecosystem functions such as being a major source of drinking water for local inhabitants and providing agriculture irrigation, flood control, and power generation [1,2]. Reservoir-ecosystem state shifts could threaten biodiversity and cause substantial losses of sustainable ecosystem goods and essential ecosystem services for human beings [3,4]. In recent years, eutrophications, and especially the formation of algal blooms, have become increasingly severe in reservoirs, and have caused periodic-rising-water quality deterioration [5,6,7]. Therefore, adequate management is essential and of utmost importance in reservoirs. A reservoir is an ideal ecological-ecosystem model to explore the physical, chemical, and biological characteristics of aquatic environments [3]. Vertical profiles of water quality are influenced by physical thermal stratification [2,3], and the drivers (e.g., temperature, light, nutrients) of algal blooms in oligotrophic drinking water reservoir shave been investigated in the past few decades. However, fewer studies have focused on the dynamics of phytoplankton and eukaryotes during mixing periods of drinking water reservoirs.

Algal blooms are widespread, and are an ongoing global problem that can affect aquatic organisms and alter water quality in aquatic ecosystems [8,9]. The dynamics of the phytoplankton population are traditionally considered to be largely influenced by abiotic variables, including nutrients concentrations, water temperature, adequate light intensity, pH values, and CO_2_ [10,11]. The biological interactions of regulating algal blooms are only just beginning to be identified. With the continuously deepening studies of freshwater-system ecological evaluation and the outbreak of mechanism of algal blooms, it is increasingly recognized that the formation of algal blooms is closely related to aquatic microbes such as bacterial and fungal communities [1,10]. These microorganisms, as a major contributor to primary productivity, play a critical role in aquatic food webs, nutrient cycling, global biogeochemical cycles, and oxygen release [12,13]. However, our knowledge about biotic factors is far from holistic, and lacks an understanding of the correlation between phytoplankton and microbial eukaryotic community composition, especially instratified drinking water reservoirs [14,15].

Phytoplankton and water eukaryotes occupy an important position in the structure and function of freshwater ecosystems, which are essential components of aquatic food webs [16]. Phytoplankton supplies oxygen and nutrients for grazers, whereas microbes decompose organic matter, recycle nutrients, and maintain the balance of ecosystems [17,18,19]. In recent years, the mutual interaction between microbes and algal bloom species has been recognized as a vital factor regulating the dynamics of microbes and algal populations [1,8,9,19,20,21,22,23,24,25,26,27,28,29]. Many interaction processes, including mutualism, algicidal effects, allelopathy, and parasitism between microorganisms and algae, can affect the formation of microbial communities [25]. Over the past few years, extensive studies have investigated bacterial [1,8,9,12,13,16,18,19,20,21,22,23,25,26,27,28,30], fungal [1,11,24,29], and zooplankton [31] community structures associated with phytoplankton dynamics using different molecular approaches, including denaturing gradient gel electrophoresis, terminal restriction fragment length polymorphism, and the Illumina Miseq DNA sequencing technique in various aquatic ecosystems such as lakes [8,9,13,16,18,19,20,31], rivers [12,28], oceans [23,24,26,30], and reservoirs [1,27]. They found that phytoplanktonis strongly linked with symbiotic microbes, which area biological disturbance to microbial communities that alter their surrounding environmental characteristics. Several recent studies have demonstrated a strong effect of phytoplankton blooms on eukaryotic plankton, which can remarkably alter the composition of eukaryotic microorganism communities [14,15,25]. Compared with bacterial, the community compositions and dynamics of eukaryotic plankton have relatively seldom been investigated in drinking water reservoirs. As primary producers and consumers, eukaryotic plankton plays an essential role in microbial food webs, which are crucial for the recycling of nutrients [14]. Therefore, their response to environmental fluctuations associated with algae may directly affect the function of aquatic ecosystems [15,32].

The ecological-network approach, a powerful bioinformatics-analysis technology, has been widely used to explore the coexistence and interaction of microbial communities [33,34] harbored in soils [35,36], sludge [37], compost [38], urban lakes [13,21,39] and marine environments [40]. In recent years, this approach has been developed and widely applied to explore the complicated mutual correlations among microbial communities [13,15,16,25], providing a unique and deep perspective on microbial interactions and ecological rules. The network contains a set of nodes or vertices that are connected in pairs by lines or edges, and it has the advantage of being able to simplify molecular ecological networks [13]. Recently, Zhang et al. [37] used Illumina Miseq DNA sequencing to reveal *nirS*-type denitrifying bacterial communities from different wastewater treatment plants, and to determine the coexistence and interaction of dominant and rare populations using network. Zhao et al. [13] investigated and compared the environmental niches of bacterioplankton in six urban lakes using network analysis, which demonstrated that co-occurrence networks varied in different seasons and that the ecological roles of cyanobacteria differed from season to season. They believed that strong co-occurrence patterns and appropriate living conditions could be critical reasons for the dominance of cyanobacteria in summer. However, from an ecological viewpoint, the dynamics and interactions of the community compositions of phytoplankton and eukaryotes during the mixing periods of drinking water reservoirs are still not comprehensively understood.

To this end, we used a high-throughput sequencing technique (Illumina Miseq DNA sequence) to investigate the composition of the water-eukaryote community in Jinpen drinking water reservoir. A traditional microscopic method was employed to analyze the abundance and composition of the phytoplankton community. Moreover, correlation-based network analysis was utilized to investigate the specific associations between these two communities. The main objectives of our present study were as follows: (1) explore the temporal variation of the phytoplankton community from October to December 2018; (2) evaluate the eukaryotic plankton community composition and structure through a high-throughput DNA sequencing technique; and (3) assess the coexistence and interaction of phytoplankton and eukaryotic microbe compositions, as well as their relationships with water-quality parameters. This study will enhance our understanding of the potential role of microbial diversity as an important driver for algal blooms in freshwater ecosystems.

## 2. Experiments

### 2.1. Study Sites and Sampling

Field sampling was conducted at Jinpen reservoir (34°42′–34°13′ N; 107°43′–108°24′ E) [2], located in the city of Xi’an, Shaanxi province, Northwest China. It is a canyon-shaped reservoir with strong thermal stratification (during summer and autumn), and serves as an important drinking water source with a daily water-supply capacity of 8.0 × 10^5^ m^3^, which accounts for 76% of the total water supply for about 8.58 million inhabitants of Xi’an [2,7,41]. The total storage capacity of Jinpen reservoir is nearly 2.0 × 10^8^ m^3^ [2,7,41]. It has a water surface area of approximately 4.55 km^2^ and a catchment area of 1418 km^2^, with a mean depth of 40 m and a maximum depth of 95 m [2,7]. Annual average rainfall, evaporation and runoff volume of Jinpen reservoir are 898 mm, 948.5 mm, and 545.22 million m^3^, respectively [2].

**Figure 1:**
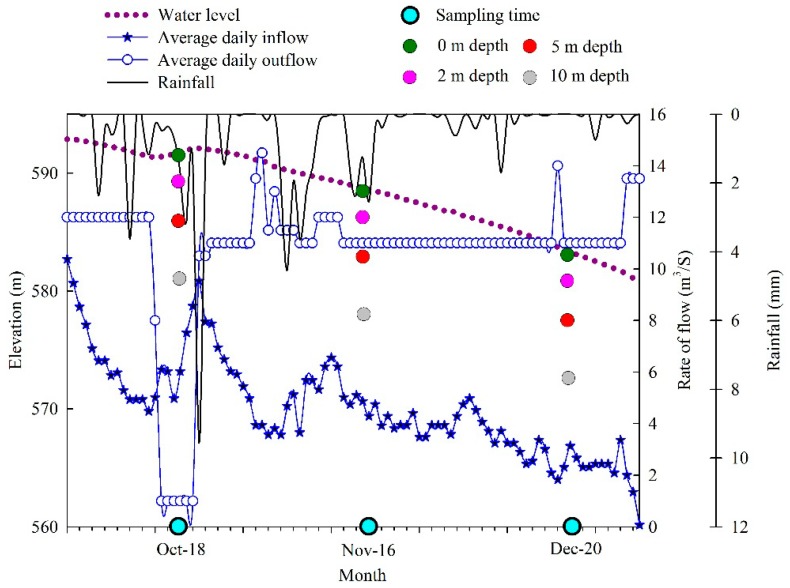
Water level, average daily inflow and outflow, rainfall, sampling times and depth in Jinpen reservoir from October to December 2018.

During the mixing periods (Figure 1), water samples were collected on a monthly basis between October and December in 2018 from different depths (0, 2, 5, 10 m) of three stations (coded as sites A, B, and C). Sites A (34°02′46″ N; 108°12′24″ E) and B (34°02′24″ N; 108°11′48″ E) are located in the main reservoir area; site A is near the intake tower for the drinking water plants, while site C (34°00′51″ N; 108°10′39″ E) is located in the upstream river area. Thus, the three sampling sites could represent the majority of cases in the entire reservoir according to the results of our research group survey [2,7]. The specific information on water level, average daily inflow and outflow, rainfall, sampling times, and depth in the Jinpen drinking water reservoir from October to December 2018 are shown in Figure 1.

All samples were collected in triplicate by a sterilized water-sampling device [2,41], stored in sterile polyethylene containers (2.0 L), kept at 4 °C, and immediately transferred to the laboratory within 24 h for further processing. Each of the samples was 2.0 L and used for chemical parameter, algal biomass, and water microbial community analyses, respectively. For microbial community analysis, 500 mL water from each sample was filtered through a 0.22 μm pore-size polycarbonate filter (Millipore, USA) and filters containing microbes were frozen at −20 °C immediately until deoxyribonucleic acid (DNA) extraction.

### 2.2. Measurement of Water-Quality Parameters

The physical characteristics of the samples, namely water temperature (WT), dissolved-oxygen (DO) concentration, pH, water turbidity, electrical conductivity (Cond.), and chlorophyll*a* (Chl-*a*) were simultaneously recorded in situ by a Multi-Parameter-Water Quality Sonde (Hydro-lab DS5, HACH Company, Loveland, CO, USA). The chemical analyses of the water samples, total nitrogen (TN), ammonium nitrogen (NH_4_^+^-N), nitrate nitrogen (NO_3_^−^-N), and total phosphorus (TP) were measured in accordance with standard methods [42,43,44]. Briefly, concentrations of NH_4_^+^-N and NO_3_^−^-N were determined following Nessler’s reagent spectrophotometry and ultraviolet spectrophotometric methods, respectively. TN and TP concentrations were quantified according to the standard UV spectrophotometric method after digestion (121 °C, 30 min; DR5000, HACH, USA) [7]. The concentrations of dissolved organic carbon (DOC) were measured with a TOC analyzer (TOC-L CPN, Japan). Concentrations of iron (Fe) and manganese (Mn) were examined using flame atomic absorption spectrometry (FAAS, AA 6800, Shimadzu, Japan) [1,2,42]. All the physicochemical analyses were performed within 48 h, and all parameters of this study were the averages of duplicate sample measurements.

### 2.3. Phytoplankton Identification and Counting

For phytoplankton analysis, 500 mL of the water sample was collected, preserved with 1% acidic Lugol’s solution, and settled for 48 h. After sedimentation for 48 h, samples were concentrated to 10 mL in order to determine algal-cell (AC) concentrations. Then, 100 μL of the concentrated sample was enumerated using a counting chamber under 400× microscope magnification after gently shaking, and recorded in units of ×10^4^ cells per liter. Each sample was generally counted twice (if the results of the two counts differed from the average by more than 15%, a third enumeration was required), then averaged to obtain a mean value for the sample [43,44]. Phytoplankton identification was performed according to Hu et al. [43], supplemented with other references [5,16,18], and identified to the lowest possible taxonomic level. All phytoplankton were enumerated and identified using a Nikon microscope (50i, Nikon, Japan).

### 2.4. Water Microbial DNA Extraction

For each site, total DNA from water microbes on the filters was extracted and purified using a Microbial DNA Kit (Omega Bio-Tek, CA, USA) according to the manufacturer’s protocol. The concentration and quality of genomic DNA were determined through spectrophotometric analysis using a Nano-Drop ND-2000 spectrophotometer (Thermo Scientific, Waltham, MA, USA) [45,46]. Then, the extracted DNA samples were stored at −20 °C prior to further analysis [19].

### 2.5. Illumina MiSeq Sequencing and Sequence Analysis

The Illumina Miseq sequencing technique was conducted to assess the water eukaryotic microbe communities. The 18S rRNA V9 region, a hypervariable area of the eukaryotic 18S rRNA gene, was amplified for analysis of the eukaryotic microorganism community using eukaryote-specific primers 1380F (5′-CCCTGCCHTTTGTACACAC-3′) and 1510R (5′-CCTTCYGCAGGTTCACCTAC-3′) [15]. All plankton DNA samples were PCR-amplified in triplicate with a PCR thermal cycler (ABI GeneAmp 9700, CA, USA). The PCR reaction mixture (30 μL) consisted of 15 μL of Phusion High-Fidelity PCR Master Mix (New England Biolabs, Beverly, MA, USA), 0.2 μM each of the forward and reverse primers, and about 10 ng of template DNA. The PCR reaction consisted of an initial denaturation for 1 min at 98 °C, followed by 30 cycles of 10 s denaturation at 98 °C, annealing at 50 °C for 30 s, elongation at 72 °C for 30 s, and a final 5 min of extension at 72 °C. The triplicate PCR products of each sample were pooled in equal quantity for gel purification, and paired-end sequencing (2 × 300 bp) was conducted on the Illumina HiSeq platform (Illumina Inc., San Diego, CA, USA) [47] at Shanghai Biozeron Bio-Technology Co., Ltd. (Shanghai, China).

### 2.6. Nucleotide-Sequence Accession Numbers

All Illumina MiSeq DNA sequence data in this study were deposited in the public NCBI Sequence Read Archive (SRA) database (http://www.ncbi.nlm.nih.gov/) under Bio-Project number: PRJNA531813 and accession numberSRP192061.

### 2.7. Statistical Analysis

Two-way ANOVA was employed with SPSS software (version 17.0, SPSS Inc., Chicago, IL, USA) using a Tukey’s HSD posthoc test (*p*-value < 0.05) to assess statistical significance among water-quality parameters, algal-cell concentration, and algal biomass under different spatiotemporal conditions.

To better understand the interactions between phytoplankton and eukaryotes, we utilized network analysis methods to analyze those two communities. In a network model, the nodes represent tax awhile the connecting edges represent correlation over time. The correlation network indicates the interactions between the abundant genera with significant (*p* < 0.05) and significantly strong (|R| > 0.8) Spearman correlations. The size of each node indicates the number of correlations. The thickness of each edge indicates the strength of the Spearman’s correlation coefficient. A network clustering diagram was generated by Cytoscape Software (version 2.8.0, http://cytoscape.org). The raw Illumina MiSeq DNA sequences were processed and checked using Quantitative Insights into Microbial Ecology (QIIME2, version 1.9.1, https://qiime2.org/). Water eukaryotic microorganism community sequence reads were filtered by the QIIME pipeline, and trimmed when they were shorter than 50 bp [15]. After assembling and quality filtering, sequences with ≥97% similarity were assigned to the same operational taxonomic units (OTUs) that were used to compute the *Chao* richness estimator (*Chao*1), Shannon diversity (*H’*), and Simpson diversity (*D*) index using the MOTHUR package (version 1.22.2, http://www.mothur.org) [1,15]. The most representative sequences for each OTU were selected, and we then used the Ribosomal Database Project (RDP) classifier (RDP Release 11.5, https://rdp.cme.msu.edu/) to annotate taxonomic information for each representative sequence [1]. Heat-map profiles were created by R Software (version 3.2.3, R core team, Vienna, Austria) [19]. To identify of the drivers of dominance in phytoplankton and eukaryote communities, redundancy analysis (RDA) was implemented on the basis of CANOCO software (version 4.5, Wageningen, The Netherlands) to explore the relationships that were consistent between eukaryotic-plankton communities and environmental variables. Prior to analysis, all water quality indicators were log^(x+1)^-transformed except for pH to meet normality and homoscedasticity using a Monte Carlo permutation test (*p*-value < 0.05).

## 3. Results and Discussion

### 3.1. Variation of Water Quality Parameters

The trends of physicochemical variables in Jinpen reservoir from October to December 2018 exhibited distinct types of temporal variation, as shown in Table 1. During the studied periods, the highest temperature was observed in October, with a maximum of 17.11 ± 0.10 °C. DO concentration declined from October to November, after which it significantly increased in December (*p* < 0.001), while pH was observed to have the same variation over the studied periods (*p* < 0.01). Generally, DOC concentration (*p* < 0.001) and conductivity (*p* < 0.001) gradually increased and peaked in November (at a depth of 5 m) and December (at a depth of 0 m) alone, with a maximum of 3.30 ± 0.14 mg/L and 157.33 ± 4.16 S/cm, respectively. The highest concentrations of Fe and Mn were both observed in December, with values of 0.038 ± 0.00 and 0.024 ± 0.00 mg/L (Table 1). A previous study conducted by Landa et al. [48] indicated that a higher water iron concentration could contribute to the proliferation of algal blooms in oceans. However, recently, Zhang et al. [1] have found that the concentration of Fe was at its lowest before the algal blooms, and that it increased markedly during the decline periods (*p* < 0.001). Compared with DOC and conductivity, concentrations of TN, NH_4_^+^-N and TP exhibited inverse trends, with the highest concentrations (as well as the highest concentrations of algal density) mainly observed in October. Recently, Su et al. [49] investigated the effect of nutrient limitation on phytoplankton growth, and found that the combination of N and P exhibited a more marked and positive influence on phytoplankton proliferation than N or P addition did independently in Spring Lake, USA. NO_3_^−^-N concentrations were in the range of 0.87 ± 0.00–0.94 ± 0.06 mg/L, and were not significantly different throughout the whole periods. Furthermore, trends of physicochemical factors were mainly consistent across all four depths, with the exception of turbidity. It was revealed that the spatial variation of all water quality parameters from October to December in Jinpen reservoir was not significant, resulting from Jinpen reservoir being in a mixed state during that period, with a similar vertical stratification of its physical and chemical factors [50]. Recently, Zhou et al. [51] conducted field research onthe impact of a water-lifting aeration system on water quality and microbial community structure in a drinking water reservoir, and found that reservoir stratification was successfully eliminated due to the artificial mixing, which strongly affected environmental variables and obviously diminished differences of water quality between the bottom and surface. Our results indicate that water quality parameters varied a lot with the changing environment, which also significantly altered the community structure of phytoplankton and eukaryotes in Jinpen reservoir. However, changing environmental conditions are in complete extrinsic factors for predicting the dynamics of phytoplankton and eukaryote communities.

### 3.2. Shifts in Algal Cell Concentration and Chlorophyll a

**Figure 2:**
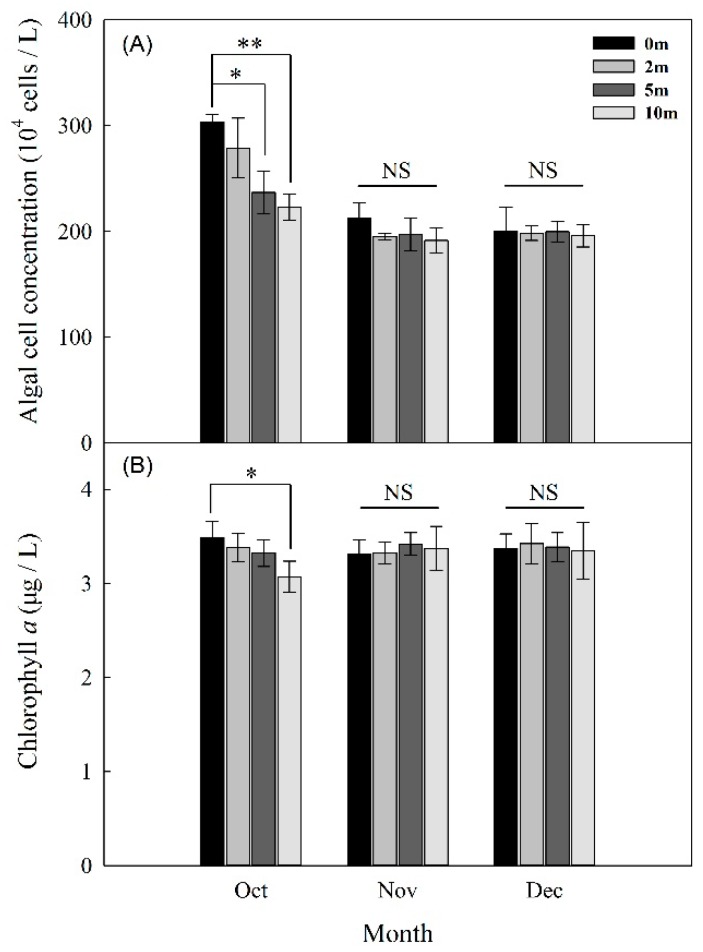
Algal cell (**A**) and chlorophyll *a* concentrations (**B**) in Jinpen reservoir from October to December, 2018, the data for each month are the means of all stations and the error bars represent the standard deviations (*n* = 3). * *p* < 0.05, ** *p* < 0.01.

Variations of algal-cell concentrations and chlorophyll *a* from October to December in 2018 are shown in Figure 2. The peaks of chlorophyll *a* (3.49 μg/L) and algal-cell concentrations (303.5 × 10^4^ cells/L) both appeared in October over the sampling period (*p* < 0.05). After that, cell concentration first decreased from 303.5 × 10^4^ to 212.5 × 10^4^ cells/L (*p* < 0.05) and finally exhibited relatively stable trends with concentrations ranging from 200.3 × 10^4^ to 212.5× 10^4^ cells/L (Figure 2A), which were generally consistent with changes in chlorophyll *a* (Figure 2B). Cell concentrations and chlorophyll *a* also exhibited significant differences (*p* < 0.05) from the depth of 0 to 10 m in October, which gradually declined (Figure 2). In contrast, both metrics exhibited less vertical variation in November and December than in October, and tended to be consistent. Results of this research indicate that phytoplankton growth in winter was not as active as in other seasonson the basis of an observed low algal biomass. Additionally, algal-cell concentrations and chlorophyll *a* were consistent with change of depth from November to December. Some explanations are as follows. On the one hand, water temperature is the most important environmental factor affecting growth, development, community structure, quantity change, and the horizontal and vertical distribution of microbes in aquatic ecosystems [11]. On the other hand, the reservoir was at the natural mixed state during the studied period, which has an extreme influence on the entire functioning of the aquatic ecosystem [50,52]. Thus, phytoplankton in reservoirs are affected when the temperature of winter is the lowest of the whole year. Bullerjahn et al. [53] investigated the dynamics and functional roles of algal and bacterial communities during winter in two seasonally ice-covered central European Great Lakes, observing that the algal biomasses of the two lakes during winter were lower than its means during a long-term summer. Diatoms, green algae, cryptophytes and chrysoflagellates were the dominant groups of algal communities, accounting for the majority of algal biomass.

### 3.3. Phytoplankton Dynamics and Community Structure

**Figure 3:**
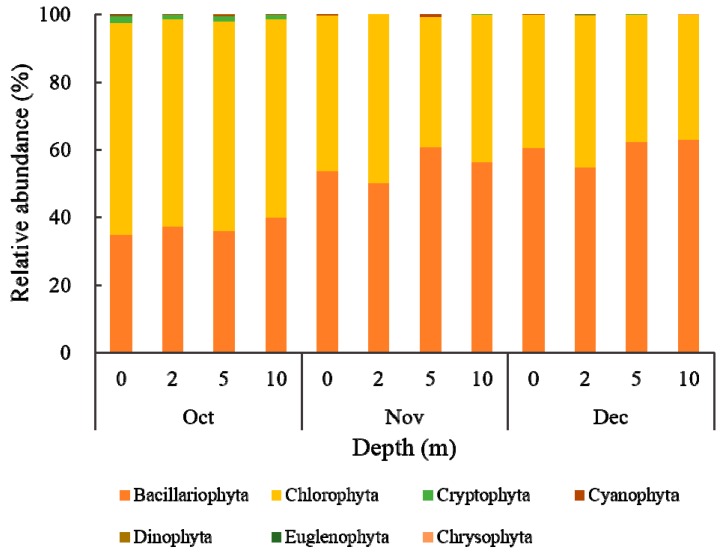
Phytoplankton community’s temporal-spatial variation at a phylum level from October to December in 2018.

In the present study, a total of 31 phytoplankton taxa were detected on the basis of morphological identification by Nikon microscopy (Figure S1). They were derived from Bacillariophyta, Chlorophyta, Cryptophyta, Cyanophyta, Dinophyta, Euglenophyta and Chrysophyta. The phytoplankton community was mainly dominated by species belonging to Bacillariophyta and Chlorophyta, accounting for more than 97% of the total phytoplankton abundance based on algal-cell concentration throughout the studied periods (Figure 3). With time, Chlorophyta decreased in relative abundance from 62.50% to 39.28% of the total phytoplankton community, whereas Bacillariophyta followed the completely inverse pattern, with a relative abundance increasing from 39.86% to 62.80%. The reservoirs showed an apparent switch from a Chlorophyta-dominated state to a Bacillariophyta taxa-dominated state. In winter, picoeukaryotes, such as common members of diatoms, green algae, and small flagellates, usually dominate the phytoplankton community [53]. Diatoms are common species in all kinds of fresh-water bodies, and widely exist in rivers, reservoirs, and lakes [54]. Previous studies have found that diatoms are very sensitive to changes in water temperature, nutrients, flow rate, predation by zooplankton, and other environmental fluctuations, which have led to corresponding variations in diatom species and densities [54]. Moreover, with advantages in turbidity and mixing environments, they are the main phytoplankton phylum found in aquatic ecosystems, and are often used as indicators for assessing ecological states such as water-quality degradation and hydrological and climate changes [12,54]. Thus, phytoplankton communities and densities were undergoing a directional change responding to environmental fluctuations during the mixing period in Jinpen reservoir.

**Figure 4:**
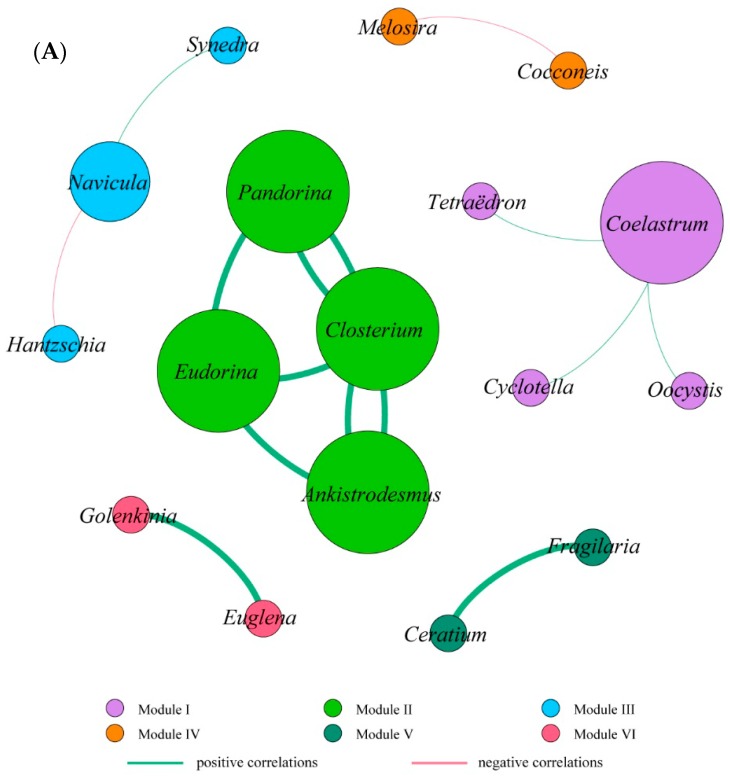

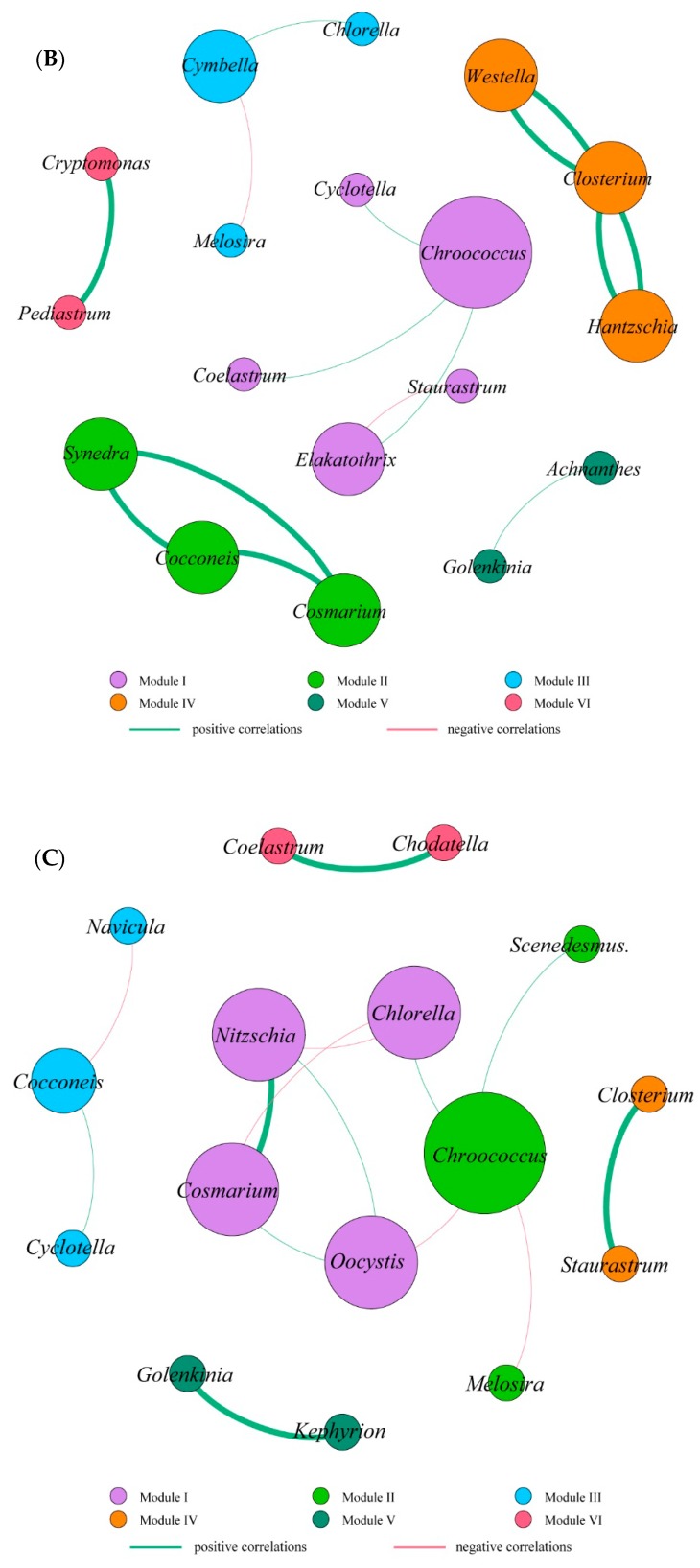
Network of co-occurring phytoplankton communities at the genus level based on a Spearman’s correlation significant analysis (*p* < 0.01) from October to December in 2018. (**A**–**C**) represents the October, November, and December sampling times, respectively. The nodes were colored according to their modularity class. Each node represents one individual algal species. The larger the node is, the more connections it has to other nodes. The green lines signify positive correlations, while the red lines signify negative correlations.

With respect to vertical variation within the phytoplankton community composition, Bacillariophyta and Chlorophyta were most dominant at depths of 5, 10, and 0, 2 m. Due to the lack of a self-regulating mechanism, Chlorophyta prefer living at the optimal light intensity in the reservoir, while diatoms are mainly distributed in the lower layer as a result of disliking light [55]. Hence, the vertical distribution of phytoplankton is closely correlated with their living habits. The Bacillariophyta and Chlorophyta communities were primarily composed of *Melosira* sp., *Cyclotella* sp., and *Chlorella* sp., respectively, and all other algal divisions accounted for less than 5% during the study period (Figure 4). However, in Lough Neagh, Bacillariophyta were mainly composed by *Fragilaria construens*, *Amphora ovalis*, and others during the winter periods, which is inconsistent with our results due to the differences of geographical distribution and water quality [56]. Seasonal variation in the phytoplankton community composition is significant. Recently, Escalas et al. [55] investigated the impact of dominant phytoplankton across four summer campaigns from 50 contrasted environmental waterbodies in the Île-de-France region, and found that Chlorophyta and Cyanobacteria were widespread in summer communities, representing most of the cases of dominance with a relative abundance of 35.5–40.6% and 30.3–36.5%, respectively. However, by investigating the effects of environmental changes on dominant phytoplankton composition, diversity, and community stability in the Římov reservoir (Czech Republic) over 32 years, Znachor et al. [57] found that the phytoplankton of the reservoir underwent a substantial compositional shift towards diatoms, and that the most significant predictors of phytoplankton-composition structure driving the overall phytoplankton assemblage were TN, inflow rate, and surface level.

A co-occurrence network that was developed and has been widely applied to reveal associations among different microbial functional groups or populations can visually show the interaction between microbial communities [13,25]. As shown in Table 2 and Figure 4, the resulting networks consisted of 17 nodes linked by 15 edges in October, 18 nodes linked by 15 edges in November, and 16 nodes linked by 14 edges in December. Overall, all nodes from different sampling times were clearly parsed into six different modules, respectively (Figure 4). Among these modules, modules I and II were the largest co-occurrence modules in the whole networks, and each module revealed different composing characteristics in a different co-occurrence network. For each co-occurrence network, most modules were relatively independent of the others, which were observed to have fewer relationships with other modules. Conversely, nodes were linked more frequently between module I and II in December. The majority of phytoplankton genera (nodes) in the same modules exhibited close positive correlations, while only a few showed negative. In total, 35 had positive correlations and 9 had negative among these significant correlations. Interspecific interactions exist widely in phytoplankton communities. Among these correlations, positive correlations in particular may indicate that microbes thrive and perform similar ecological functions under the same environment [58]. The obvious differences of the three different networks indicated that correlations between phytoplankton could adjust with time. The main reasons for the seasonal variation of the networks are that the phytoplankton community composition varies over time, and that the dynamics of the environmental parameters can affect the relationships between algal species. For examples, species that share similar ecological niches probably exhibit competitive or mutually exclusive roles in resource shortages, but may reveal positive interactions in resource-rich environments [13]. Some studies proposed that the community structure of phytoplankton is potentially regulated conjointly by both extrinsic and intrinsic drivers deriving from the surrounding environment and interspecific interactions between two species. Recently, Yang et al. [59] explored the mechanisms of phytoplankton community dynamics and demonstrated that interspecific interactions were more important than extrinsic factors in shaping phytoplankton community structure, with a maximum value of 48% in random-forest-based models. Furthermore, they found the key stone species of *Oscillatoria granulate* were positively correlated with *Euglena pisciformis*, *Trachelomonas* and *Phacus*, but negatively correlated with *Chlorella*.

### 3.4. Microbial Eukaryotic Community Composition

**Figure 5:**
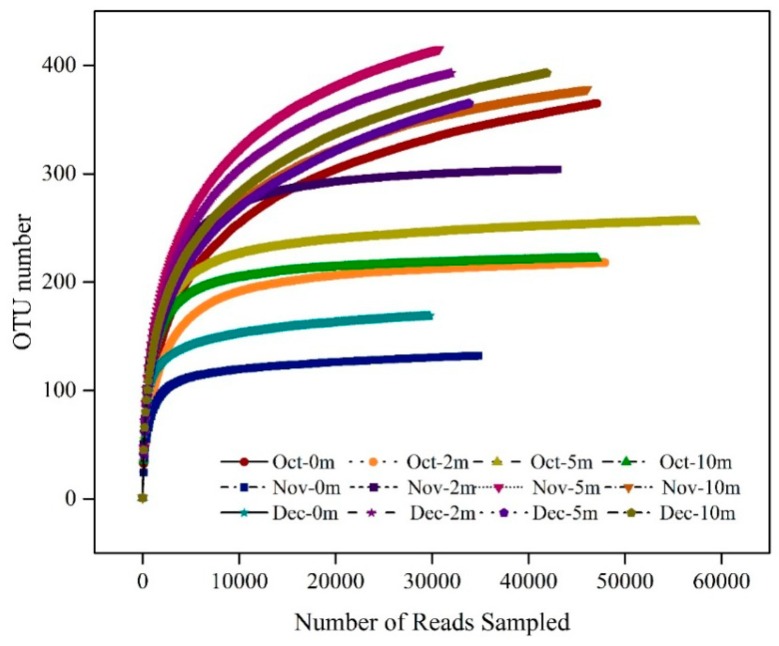
Rarefaction curves of the operational taxonomic unit (OTU) numbers at 97% similarity for temporal-spatial distribution of the eukaryotic community in Jinpen reservoir from October to December 2018.

Rarefaction curves of OTU number sat a 97% similarity box plot revealed that many reads were sampled (Figure 5). On the basis of the Illumina Miseq high-throughput sequencing, a total of 503,848 high-quality sequences were yielded from 12 samples, with an average length of 134 bp (triplicates) after quality trimming (Table 3 and Figure 5). Miseq sequencing obtained a total of 3610 operational taxonomic units (OTUs) with 97% similarity. The coverage of eukaryotic microbial communities from 12 samples reached 99%, indicating that the sequencing depth of eukaryotes had reached a high level with high data reliability. There were 203,565,159,021,141, and 262 read numbers from October to December, and the average read numbers for each month were 50,891,39,755, and 35,316, respectively. The highest OTUs were observed at a depth of 5 m in November, and the lowest were at a depth of 0 m in December (415 and 132, respectively). Similarly, on the basis of the Illumina Miseq sequencing technique, Zhang et al. [41] investigated the vertical distribution of bacterial communities during thermal stratification in a drinking water reservoir and observed that the highest and lowest OTUs were found in 5 and 0.5 m, respectively.

To explore the dynamic of water eukaryotic community structures, we utilized species-richness estimates (*Chao* 1) and diversity indices (Shannon, Simpson). Diversity estimators *Chao* 1, Shannon, and Simpson clearly varied between months and depths (Table 3). Overall, *Chao* 1 indices were lowest in October and highest from November to December, ranging from 143 to 476. Moreover, the *Chao* 1 richness index was higher than the OTU value, indicating that more unidentified sequences are still to be studied in the reservoir [50]. Variations of Shannon diversity (*H’*) were analogous to the *Chao* 1 index. As shown in Table 3, the highest Shannon diversity indices (*H’*) and the lowest Simpson diversity indexes (*D*) were both observed in November at a depth of 2 m, with values of 4.3 and 0.026, respectively, indicating the highest diversity and most even taxon distribution of eukaryotic communities during this period. Spatial variations of eukaryotic community diversity generally also exhibited surface water that was lower than the other layers. Previously, Zhang et al. [41] reported that the water microbial community *Chao* 1 index ranged from 407 to 506, changing with depth. Additionally, Shannon diversity (*H’*) was lower in the surface layer and higher in the bottom. By investigating the impacts of thermal stratification on the bacterial community with the methods of BIOLOG and 454 pyrosequencing, Yang et al. [50] also found that bacterial community diversity in the hypolimnion was higher than that in the epilimnion, indicating that stratification significantly affected microbial communities.

**Figure 6:**
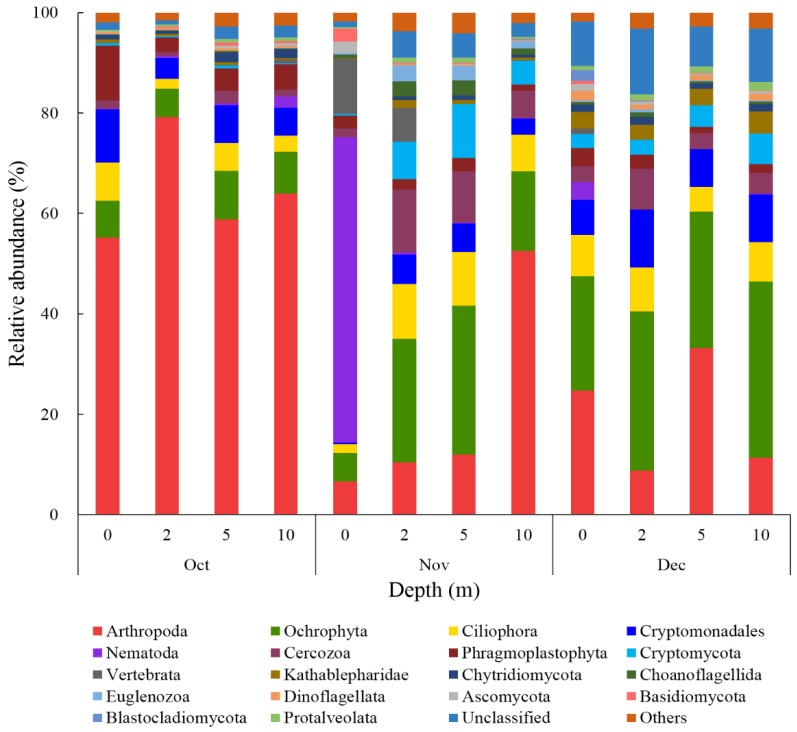
Eukaryotic microorganism community compositions at phylum levels (top 18) in samples from October to December in 2018.

The eukaryotic community at the phylum level distinctly varied during the studied periods in Jinpen reservoir. Figure 6 shows an overview of the eukaryotic community composition. The 3610 operational taxonomic units (OTUs) with 97% similarity were mainly classified into 20 phyla: Arthropoda, Ochrophyta, Ciliophora, Cryptomonadales, Nematoda, Cercozoa, Phragmoplastophyta, Cryptomycota, Vertebrata, Kathablepharidae, Chytridiomycota, Choanoflagellida, Euglenozoa, Dinoflagellata, Ascomycota, Basidiomycota, Blastocladiomycota, Protalveolata, Unclassified and Others (Figure 6). Different from the variation of algal community composition and abundance, eukaryote biodiversity increased with time, and species distribution was more even (Figure 6). From October to December, Arthropoda (6.63% to 79.19%), Ochrophyta (5.60% to 35.16%), Ciliophora (1.81% to 10.93%), and Cryptomonadales (0.25% to 11.48%) were the most dominant eukaryotes in common, which contributed over 50% of the total eukaryotic community (except for the point of 0 m in November). From those taxa, arthropod abundance declined significantly over time, especially at the point of 0 to 5 m in November, with an average decrease of 55.55% (Figure 6), while algal density was also markedly reduced during this period. Arthropoda, as a kind of zooplankton, grow by feeding on algae, organic particles, and bacteria, and some even have algae as their sole source of nutrition. Thus, to a certain extent, arthropod growth might be affected by the decrease of their food sources [31]. Recently, research observed that a relative abundance of arthropods declined over time in a subtropical reservoir, and that they were one of the major contributors to eukaryotic plankton community rearrangements [15]. In addition, Nematoda peaked in November (at a depth of 0 m), with a maximal relative abundance of 60.87% (Figure 6), becoming the most abundant and unique species during this period, which was rare in other periods. At the same time, the algal-cell concentration sharply decreased, with a maximal reduction of 91 × 10^4^ cells/L. Special variation of Nematoda in November might be associated with the sharp decrease of algal cell concentration at a depth of 0 m, which meant that a marked decrease of algae might boost the active growth of Nematoda. Under the same condition, differences between Arthropod and Nematoda dynamics possibly resulted from two aspects: on the one hand, there are different resource-utilization characteristics and abilities of these two eukaryotes to adapt to particular ecosystems; on the other hand, there are interrelationships between different eukaryotes in terms of predation, competition, and parasitism [11]. Cercozoa dominated from November to December, with a maximal relative abundance of 12.50% in November (at a depth of 2 m). Similarly, as the common dominant taxon, Ciliophora were also observed with a maximal relative abundance (10.93%) in November (at a depth of 2 m). Ciliophora and Cercozoa are widely distributed and ubiquitous in lakes [11,14,32,60,61] and reservoirs [15,62], and they are important consumers of algae and bacteria, which occupy a pivotal position in aquatic food webs by transferring nutrients to higher trophic levels [61]. Nematoda, Ciliophora, and Cercozoa are also affiliated with zooplankton, inducing a decrease of algal density by predation and altering the structure of phytoplankton communities by selecting specific algae [31]. Several studies have reported that variations in zooplankton species distribution and abundance may alter ecosystems via cascading effects, and found that climate variability can be indicated by zooplankton [55,56,63]. Freshwater fungi are ubiquitous and diverse, occupying an important position as saprotrophs or parasites in the function of freshwater ecosystems [24]. In this study, five fungus phyla were observed in the eukaryotic community: Cryptomycota, Chytridiomycota, Ascomycota, Basidiomycota, and Blastocladiomycota. Compared with other fungi, the abundance of Cryptomycota significantly increased in November and December, reaching a maximum of 10.70% (at a depth of 5 m) in November, whereas algal density decreased during this period. Cryptomycota, a newly discovered type of fungus, is widely found in water environments and usually parasitizes algae or other fungi, absorbing nutrients through phagocytosis and thereby directly affecting the growth of hosts [64,65]. Hence, results in our study partly confirmed that there might be a saprophytic interaction between Cryptomycota and algal decomposition. Recently, Cryptomycota were also identified by a study conducted by Zhang et al. [66] exploring the relationships between axenic *Microcystis aeruginosa* and aquatic microbes by using a laboratory co-culture system. They found that abundance of Cryptomycota and other eukaryotes decreased after co-culturing.

**Figure 7:**
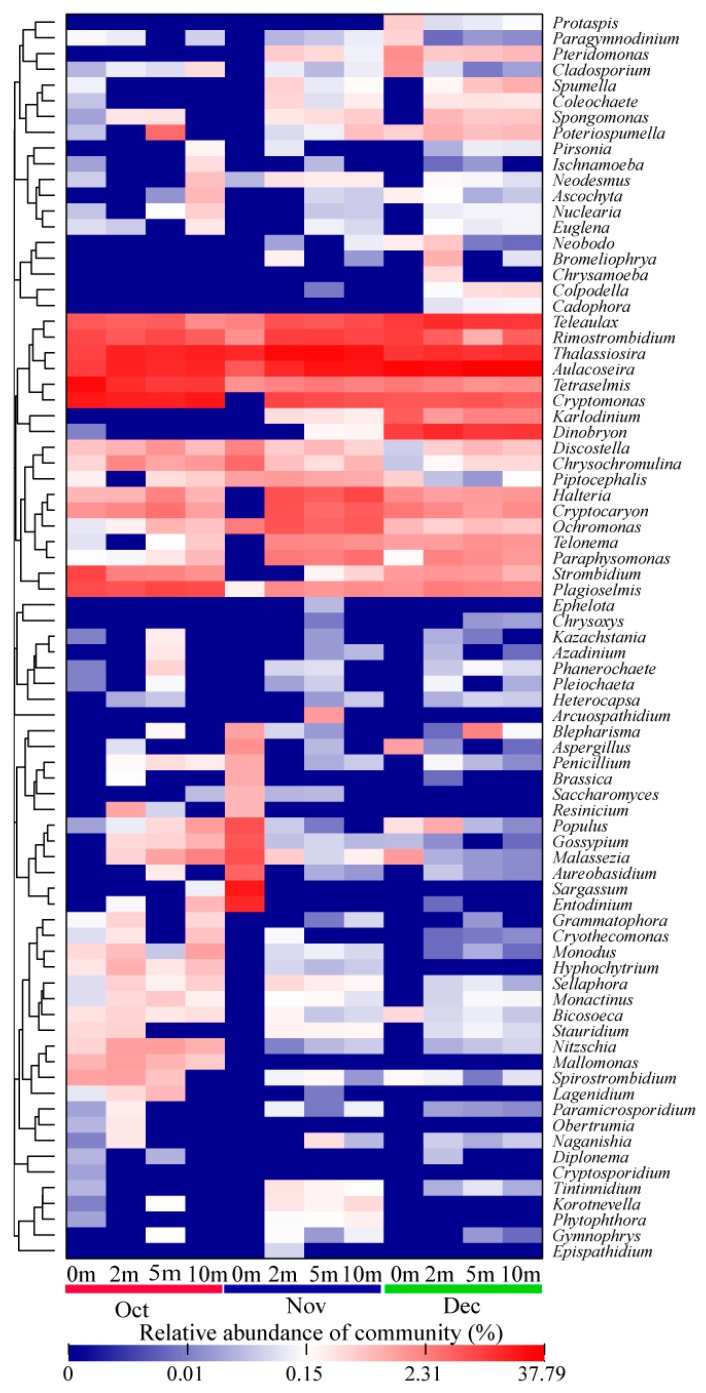
A color-scale heat map showing 79 predominant 18S rRNA gene-based eukaryotic sequences classified at the genus level. Red colors indicate higher abundance; blue colors indicate lower abundance.

To further investigate the eukaryotic community, a heat-map fingerprint with 79 representative eukaryotic species was generated at the genus level, as shown in Figure 7. Consistent with the variation of phylum level, the eukaryotic community composition in Jinpen reservoir exhibited distinct patterns at the genus level, which became more diverse and different over time. *Aulacoseira* and *Thalassiosira* were the common genera in the entire sampling time, and contributed to a higher relative abundance for the eukaryotic community with a sum of relative abundance ranging from 17.93% to 49.56%. In October, *Tetraselmis* and *Cryptomonas* were the most abundant genera in addition to the common genera. From these two taxa, *Tetraselmis* reached its maximal relative abundance of 25.82% at a depth of 0 m, while *Cryptomonas* was absolutely dominant at depths from 2 to 10 m (16.24% to 20.29%). *Strombidium* was another abundant genus in October appearing at a depth of 0 m witha relative abundance of 8.28%, which was rare atother sampling times. In November, *Sargassum* contributed to the highest relative proportions in the community at a depth of 0 m, with a relative abundance of 18.86%, and was closely followed by *Entodinium* (14.20%). *Bodo*, *Rimostrombidium*, and *Halteria* dominated at depths of 2, 5, and 10 m and accounted for 8.92%, 8.55%, and 7.57%, respectively (with the exception of *Aulacoseira* and *Thalassiosira*). Consistent with previous observations, Alveolata comprising the specific genera *Halteriagrandinella* and *Rimostrombidiumlacustris* was found as the second most important taxon, dominating in the day-4 clone library during a Mesocosm experiment [14]. In December, *Teleaulax* and *Dinobryon* were other abundant genera after *Aulacoseira* and *Thalassiosira*, with an average relative abundance of 11.33% and 11.17%, respectively. *Teleaulax* belongs to Cryptophyta, which was also detected by Mikhailov et al. [60] during spring algal blooms in Lake Baikal. It contributed the highest proportions of 15% for total eukaryotic reads, and was positively correlated with *Cryptococcus*. Mikhailov et al. also observed *Halteria* ranging from 0.1% to 23%, with a higher betweenness centrality of 0.105 in the co-occurrence network of microbial eukaryotes.

### 3.5. Relationships between Phytoplankton and Eukaryotic Communities Explored by the Network Approach

**Figure 8:**
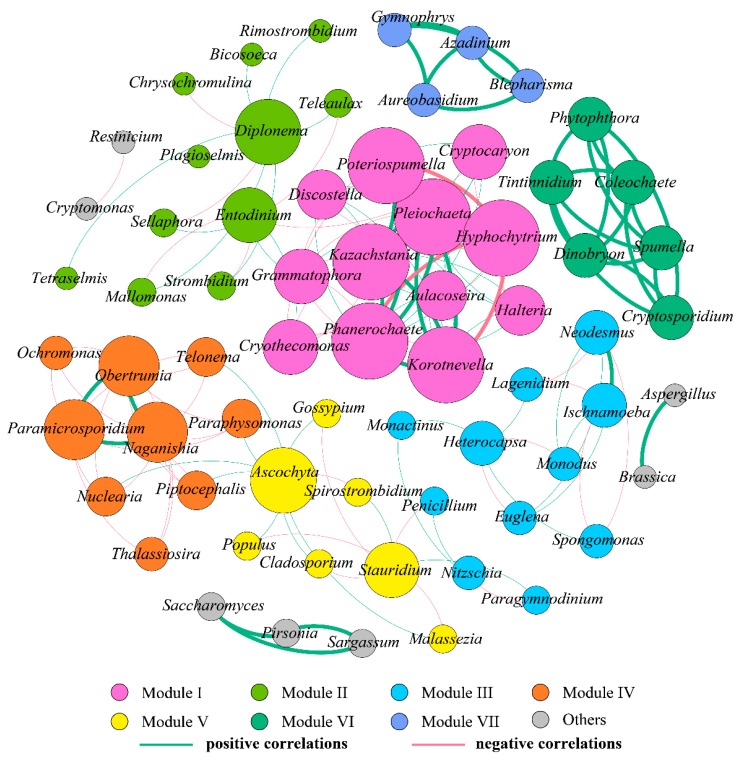
Coexistence and interaction of eukaryote community compositions at the genus level based on the 18S rRNA sequence and associated with a water sample in October 2018. The nodes were colored according to modularity class. Each node represents one individual eukaryotic species. The larger the node is, the more connections it has to other nodes. The green lines signify positive correlations, while the red lines signify negative correlations.

**Figure 9:**
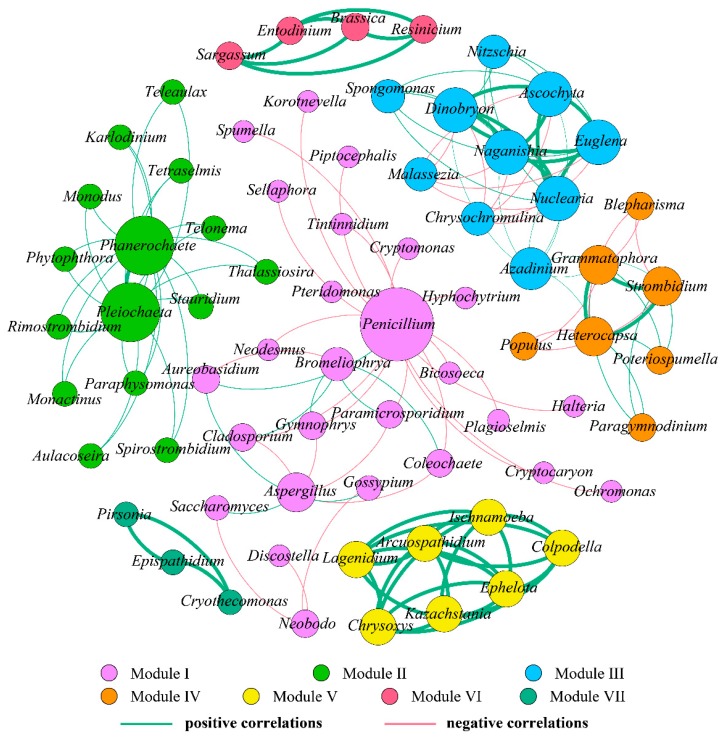
Coexistence and interaction of eukaryote community compositions at the genus level based on the 18S rRNA sequence and associated with a water sample in November 2018. The nodes were colored according to modularity class. Each node represents one individual eukaryotic species. The larger the node is, the more connections it has to other nodes. The green lines signify positive correlations, while the red lines signify negative correlations.

**Figure 10:**
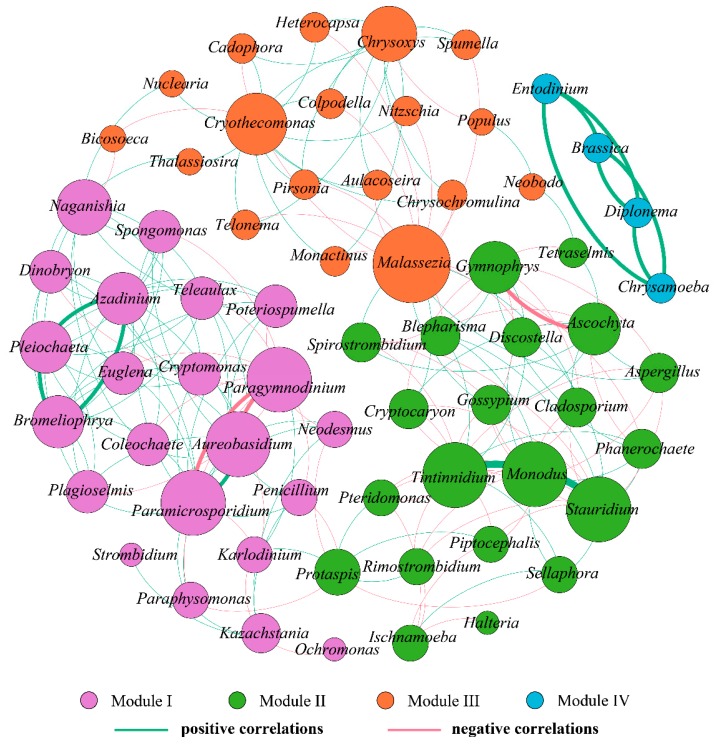
Coexistence and interaction of eukaryote community compositions at the genus level based on the 18S sRNA sequence and associated with water sample in December 2018. The nodes were colored according to modularity class. Each node represents one individual eukaryotic species. The larger the node is, the more connections it has to other nodes. The green lines signify positive correlations, while the red lines signify negative correlations.

Co-occurrence networks provide synthetic and clear representations of the microbial community for a better understanding of its structure and function [25,55]. Here, we assessed the co-occurrence patterns of eukaryotic organisms at the genus level from October to December to explore interactions between different eukaryotes. Co-occurring eukaryotic genus networks showed that community composition significantly variedin the three months. As shown in Table 4 and Figure 8, Figure 9 and Figure 10, the resulting networks consisted of 67 nodes linked by 147 edges in October, 72 nodes linked by 144 edges in November, and 65 nodes linked by 204 edges in December. The observed link numbers increased, reaching their maximum in December, whereas node numbers were similar in the three months. Network density was highest in December, while the October and December networks exhibited lower and similar density. The indices of average clustering coefficient and average path length represent the size of the networks [55], which decreased and increased from October to December, respectively, and reached their minimum and maximum in December (Table 4).

In October, all nodes were clearly parsed into eight different modules (Figure 8). Among these modules, modules I to VI, as the largest co-occurrence modules, dominated in the whole network, containing 12, 11, 11, and 9 nodes (genera), respectively, witheach module exhibiting different composing characteristics. The connected nodes with a larger size (e.g., *Pleiochaeta*) had higher significant negative or positive associations with other taxa (genera). In module I, *Hyphochytrium* was observed to have significant negative correlations with *Poteriospumella*, *Phanerochaete*, and *Korotnevella*, while significant positive correlations existed among *Pleiochaeta*, *Poteriospumella*, *Kazachstania*, *Phanerochaete*, *Korotnevella*, and *Aulacoseira*. Modules VI and VII and others were independent, and observed to have fewer relationships with other modules. Eukaryotes from these three modules revealed a significant positive correlation with one another. However, eukaryotes from module IV mostly showed significant a negative correlation with one another, and only three significant positive correlations were observed among *Naganishia*, *Obertrumia*, and *Paramicrosporidium*.

In November, all nodes were clearly parsed into seven different modules, of which modules I–III dominated in the network, accounting for 36.11%, 20.83%, and 13.89% of the whole network, respectively (Figure 9). In module I, notably, *Penicillium* was observed to have more significant correlations with other eukaryotic genera, with the highest degree (number of connections) of 19. Among these correlations, 18 were negative and only one was positive (with *Bromeliophrya*), indicating that *Penicillium* negatively affected the growth of the majority of other genera, or that there might be an antagonistic and competitive [36], or saprophytic [14], association between *Penicillium* and these genera. Correlations from modules II and V–VII were all positive, and positive correlations from modules V–VII were especially significant, indicating that the eukaryotic genera from these modules might arise from similar niches or mutualism [60,61,66]. Additionally, in module II, *Phanerochaete* was observed to have the highest positive correlations with other eukaryotic genera (adegree of 20), followed closely by *Pleiochaeta* with a degree of 13. *Naganishia*, *Dinobryon*, *Nuclearia*, *Ascochyta*, and *Euglena* from module III were significantly and positively correlated with each other, which is consistent with the significant association among *Grammatophora*, *Strombidium*, and *Heterocapsa* from module IV.

In December, all nodes were clearly parsed into four different modules, which was the lowest during the studied period (Figure 10). Modules I–III comprised 22, 21, and 18 nodes (genera), respectively, when the module contained only four nodes. In module I, significant negative correlations with *Aureobasidium* and *Paramicrosporidium* were observed for *Paraphysomonas*, while significant positive correlations were observed between *Aureobasidium* and *Paramicrosporidium*. Likewise, in module II, *Monodus* was significantly and positively linked with *Tintinnidium* and *Stauridium*, whereas a distinct negative association existed between *Ascochyta* and *Gymnophrys*. In module III, *Malassezia* had the highest degree (18), but was negatively correlated with numerous eukaryotic genera. In contrast, *Cryothecomonas* was observed to have the highest positive correlations with other eukaryotic genera (a degree of 15). It was followed closely by *Chrysoxys* with a degree of 12. Module VI was relatively independent while other modules were linked more frequently, and genera from module VI all exhibited significant positive correlations with one another.

To summarize, the characteristics of co-occurring eukaryotic genera networks further showed that community composition strongly varied over time. Eukaryotes exhibited significant and predominantly positive or negative correlations between each other, which were specific to time. Positive correlations might arise from similar niches or mutualism while negative correlations might reflect a non-overlapping niche or antagonism [61]. Analysis of macro-ecological networks shows that co-occurrences may be a better indicator of niche preferences than biotic interactions [11]. Recently, Xue et al. [15] employed co-occurrence network analysis to investigate the relationships of abundant and rare planktonic eukaryotes in a subtropical reservoir, and found that dominant taxa were mainly derived from rare species that may play vital roles in regulating the ecological function in aquatic systems as keystone species. The authors also observed that rare and non-rare taxa exhibited synergistic effects that might contribute to maintaining the stability and resilience of eukaryotic communities and ecological function. Mikhailov et al. [60] found that species from the microbial metacommunity occupied positive correlations and were highly interconnected with one another within a specific domain. Positive associations between bacteria and eukaryotes may be used as evidence of mutualistic interactions, whereas negative associations may indicate competition.

### 3.6. Effect of the Environmental Factors on Communities

**Figure 11:**
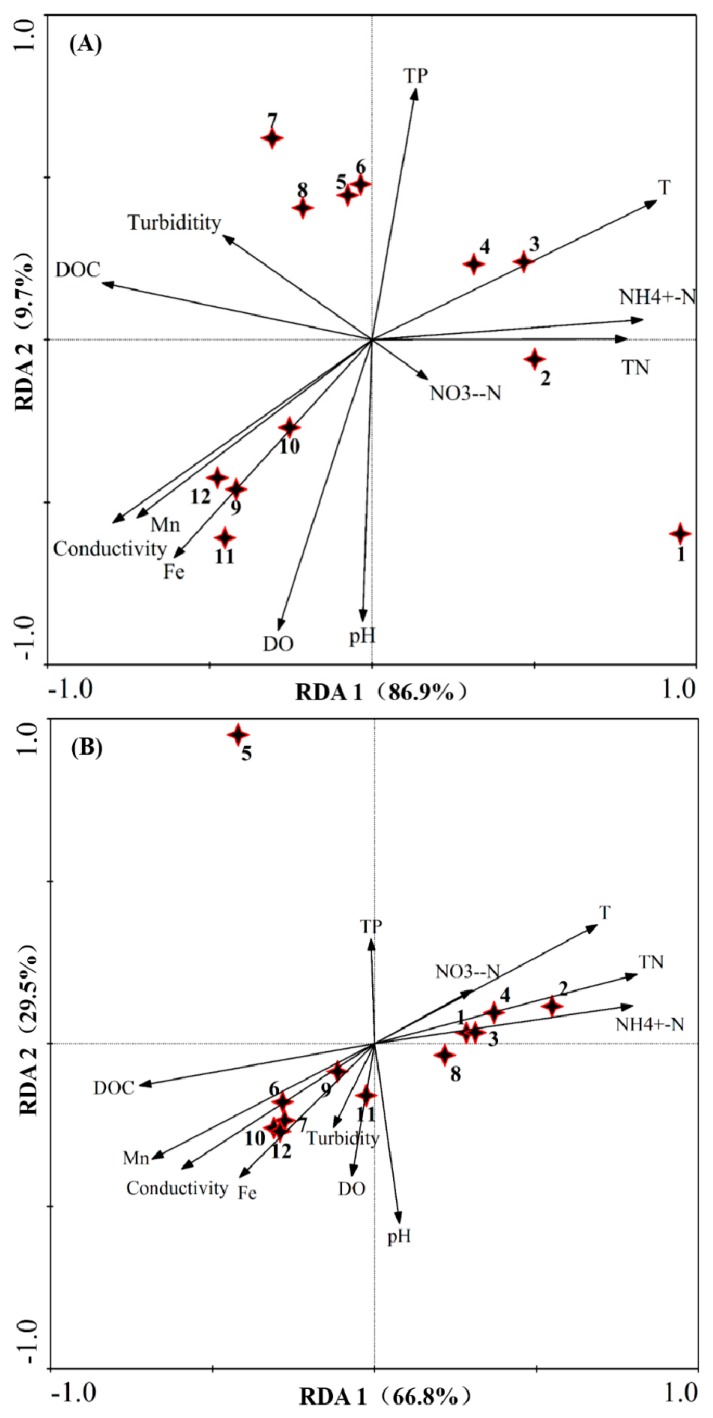
Redundancy analysis (RDA) of algal (**A**) and eukaryotic (**B**) communities in Jinpen reservoir from October to December 2018. Stars represent sampling points and 1,2, 3, 4, 5, 6, 7, 8, 9, 10, 11, and 12 represent Oct.—0 m, Oct.—2 m, Oct.—5 m, Oct.—10 m, Nov.—0 m, Nov.—2 m, Nov.—5 m, Nov.—10 m, Dec.—0 m, Dec.—2 m, Dec.—5 m, and Dec.—10 m, respectively. For the algal community, RDA1 explains 86.9%, and RDA2 explains 9.7% of the total variance. For the eukaryotic community, RDA1 explains 66.8%, and RDA 2 explains 29.5% of the total variance. The primary factors for the variables of the water quality data are represented by arrows. DO = dissolved oxygen; TN = total nitrogen; TP = total phosphorus; DOC = dissolved organic carbon.

To better understand the effects of water quality on phytoplankton and eukaryote community diversity, multivariable statistics from redundancy analysis (RDA) were employed to investigate the relationships between water-quality parameters and compositions of algal and eukaryotic communities on the basis of microscopy analysis and Illunina MiSeq sequencing. RDA results revealed that distinct phytoplankton and eukaryote communities were detected during the studied periods in the Jinpen drinking water reservoir. As shown in Figure 11, environmental parameters explained 96.6% variance of phytoplankton communities and 96.3% variance of eukaryote communities, respectively. Phytoplankton community structure was significantly influenced by water temperature, NH_4_^+^-N, TN, TP, DOC, conductivity, Mn, Fe, DO, and pH. The most important determinants of eukaryotic community structure were TN, NH_4_^+^-N, water temperature, DOC, Mn, conductivity, and pH among all the physic-chemical parameters. Moreover, TN, NH_4_^+^-N, and water temperature exhibited a reverse correlation relative to DOC, Mn, conductivity, and pH. This suggests that water quality significantly affects the variability of phytoplankton and eukaryote community compositions, particularly TN, NH_4_^+^-N, water temperature, DOC, Mn, conductivity, and pH, which were common factors in controlling population variation. However, DO, TP, and Fe were the only influential factors on the structure of the phytoplankton communities. A similar study conducted by Chen et al. [14] indicated that the microbial eukaryotic community composition was mostly correlated with changes of pH, DO, and DOC concentrations, which is generally consistent with our results.

By investigating the variations of phytoplankton community structure over two years (a wet year and a dry year), Qiu et al. [45] observed that nitrogen fluctuations caused by rainfall were more significant than other environmental parameters in the Zhoucun reservoir, which is one of the crucial factors responsible for the dynamics of phytoplankton growth. In addition, they found that water temperature, thermal stratification, and light were considerable driving factors for shaping the phytoplankton community. Recently, Yang et al. [4] found that combined disturbance events such as water-level fluctuations, human activities, climate change, and rainfall exert a complex and diverse impact on phytoplankton communities across space and time compared to single disturbances. They observed that phytoplankton community dynamics in two subtropical reservoirs over the past six years reflected distinct and reversible patterns (from a cyanobacteria-dominated state to non-cyanobacterial-taxa-dominated state) under multiple environmental-disturbance conditions. From those disturbances, water temperature and rainfall strongly affected cyanobacterium biomass, while nutrients and water level, as the main driving factors, had great impacts onthephytoplankton community structure. Moreover, the impact of the disturbances varied in the taxon and organizational levels. However, Bullerjahn et al. [53] recently observed that winter phytoplankton communities in ice fractions were photo-synthetically active. Additionally, the similarities of ice-derived microbial communities in these two totally different lakes suggest that the conditions for ice formation chose common community compositions. Capo et al. [32] demonstrated that modules comprising Dinophyceae and unclassified Alveolata were significantly related to air temperature in two lakes that were exposed to varying degrees of phosphorus enrichment. Compared with our study, these inconsistent results indicate that microbial community compositions in an aquatic system are affected by a variety of environmental factors and are altered by changes in local conditions, leading to totally different community structure variation trajectories.

In conclusion, our results demonstrated that phytoplankton and eukaryotes in the water column were both affected by changing environmental conditions, and that they responded accordingly. At the same time, such extrinsic factors deriving from the surrounding environment are incomplete for explaining the dynamics of phytoplankton and eukaryote communities. Most importantly, intrinsic feedback mechanisms and interspecific interactions play critical roles in a community’s response to variations in external factors [10]. Our results revealed that relationships between phytoplankton and eukaryotes were complicated and important. The mutual interactions existed widely and exerted a strong impact on shaping phytoplankton and eukaryote assemblage. For example, Ciliophora and Cercozoa, which are important consumers of algae and bacteria, were dominant in the eukaryotic community. Phytoplankton blooms can boost the growth rate of microbes [15]. Thus, the increase of these taxa most likely resulted from the increase of their prey or food. Results in our study also exhibited proof that the growth of phytoplankton is closely related to fungus. For example, Cryptomycota had a saprophytic interaction with phytoplankton. Furthermore, *Penicillium* exhibited higher connections with other eukaryotic genera that were negatively related to others such as *Cryptomonas*. These negative correlations indicated that there might be antagonistic or saprophytic relationships between *Penicillium* and these genera. Conversely, our co-occurrence network also showed some negative associations among eukaryotes. *Phanerochaete*, *Pleiochaeta*, *Cryothecomonas*, and *Chrysoxys* positively correlated with other genera. Our results provide a deeper insight into the dynamics of phytoplankton and eukaryote communities in reservoirs from a new perspective. Given our observations, we recommend that future studies focus on understanding the correlations of phytoplankton and eukaryote communities through long-term and extensive investigations in order to fully understand their intrinsic mechanisms and relative importance in affecting microbial community dynamics.

## 4. Conclusions

In summary, results of this study proved that variations of phytoplankton and water eukaryotes were closely associated with each other during winter in the Jinpen drinking water reservoir. Significant spatial-temporal changes were revealed in the composition of the eukaryotic and phytoplankton communities. The co-occurrence of phytoplankton indicated that the community structure varied remarkably over time. Moreover, Bacillariophyta and Chlorophyta were the most abundant taxa, with a total relative abundance of more than 97% throughout the studied periods, which were primarily composed of *Melosira* sp., *Cyclotella* sp., and *Chlorella* sp., respectively. Illumina Miseq sequencing suggested that eukaryote biodiversity gradually increased and species distribution became more even over time. Arthropoda (6.63% to 79.19%), Ochrophyta (5.60% to 35.16%), Ciliophora (1.81% to 10.93%), and Cryptomonadales (0.25% to 11.48%) were the most dominant eukaryotes in common, contributing over 50% of the total eukaryotic community. The abundance of arthropod was significantly reduced at the point of 0 to 5 m from October to November, with an average decrease of 55.55%, while algal density significantly declined at the same time, indicating a negative association between arthropod and phytoplankton. Nematoda and Cercozoa peaked in November with maximal relative abundances of 60.87% and 12.50%, respectively. Cryptomycota, a newly discovered type of fungus, was special among the most dominant eukaryotes; when the number of algae decreased, the abundance of Cryptomycota increased, suggesting that there might be a saprophytic interaction between Cryptomycota and phytoplankton decomposition. The co-occurrence network analysis showed that there existed significant correlations between eukaryotic and phytoplankton community composition, suggesting that eukaryotes respond to variations in the dynamic structure of phytoplankton community. For instance, *Penicillium* was observed in the majority of significant negative correlations with other eukaryotic genera, which indicates that *Penicillium* negatively affects the growth of other genera and that there might be an antagonistic and competitive (or saprophytic) association between them. In addition, RDA results demonstrated that total nitrogen, ammonia nitrogen, water temperature, DOC, Mn, conductivity, and pH contributed the most to variations of phytoplankton and eukaryotic microorganism communities. Furthermore, the DO, TP, and Fe factors can also significantly alter the structures of phytoplankton communities. These conclusions confirm that intricate and vital mutual interactions exist between phytoplankton and eukaryotes in freshwater ecosystems. On this basis, more studies are needed to further investigate the ecological functions of water microbes in drinking water reservoirs.

## Figures and Tables

**Table 1:** Water quality parameters of Jinpen reservoir measured from October to December in 2018.

Month	Depth (m)	T (°C)	DO (mg/L)	Turbidity (NTU)	Conductivity (S/cm)	pH	DOC (mg/L)	TN (mg/L)	NO_3_^−^N (mg/L)	NH_4_^+^-N (mg/L)	TP (mg/L)	Fe (mg/L)	Mn (mg/L)
October	0	17.11 ± 0.10	8.57 ± 0.10	0.23 ± 0.06	142.67 ± 2.52	7.69 ± 0.02	2.09 ± 0.09	1.33 ± 0.04	0.90 ± 0.02	0.09 ± 0.03	0.02 ± 0.00	0.013 ± 0.00	0.005 ± 0.00
	2	16.93 ± 0.21	8.49 ± 0.11	0.47 ± 0.07	142.67 ± 2.52	7.67 ± 0.03	1.92 ± 0.07	1.27 ± 0.01	0.88 ± 0.01	0.09 ± 0.02	0.02 ± 0.00	0.014 ± 0.00	0.003 ± 0.00
	5	16.78 ± 0.24	8.42 ± 0.18	0.70 ± 0.00	142.33 ± 2.08	7.65 ± 0.04	2.20 ± 0.30	1.36 ± 0.03	0.88 ± 0.01	0.10 ± 0.02	0.03 ± 0.00	0.013 ± 0.00	0.004 ± 0.00
	10	16.78 ± 0.26	8.42 ± 0.21	1.03 ± 0.00	142.33 ± 2.08	7.63 ± 0.04	1.98 ± 1.33	1.39 ± 0.09	0.89 ± 0.00	0.10 ± 0.02	0.02 ± 0.00	0.012 ± 0.00	0.004 ± 0.00
November	0	14.44 ± 0.07	8.06 ± 0.18	0.62 ± 0.00	146.33 ± 0.58	7.55 ± 0.05	2.99 ± 0.20	1.18 ± 0.09	0.88 ± 0.02	0.06 ± 0.01	0.03 ± 0.00	0.008 ± 0.00	0.008 ± 0.00
	2	14.45 ± 0.06	8.05 ± 0.22	0.80 ± 0.06	146.00 ± 1.00	7.61 ± 0.04	2.69 ± 0.50	1.13 ± 0.03	0.87 ± 0.01	0.06 ± 0.01	0.03 ± 0.00	0.008 ± 0.00	0.008 ± 0.00
	5	14.45 ± 0.06	8.03 ± 0.25	1.13 ± 0.07	146.33 ± 0.58	7.60 ± 0.03	3.30 ± 0.14	1.18 ± 0.01	0.87 ± 0.00	0.07 ± 0.01	0.03 ± 0.00	0.009 ± 0.00	0.010 ± 0.00
	10	14.46 ± 0.07	8.01 ± 0.22	1.57 ± 0.14	146.00 ± 1.00	7.57 ± 0.02	3.28 ± 0.11	1.20 ± 0.06	0.90 ± 0.03	0.06 ± 0.01	0.03 ± 0.00	0.013 ± 0.00	0.010 ± 0.00
December	0	11.07 ± 0.20	8.99 ± 0.61	0.17 ± 0.12	157.33 ± 4.16	7.72 ± 0.05	3.06 ± 0.39	1.21 ± 0.10	0.94 ± 0.06	0.06 ± 0.01	0.01 ± 0.00	0.038 ± 0.00	0.016 ± 0.00
	2	11.02 ± 0.16	9.09 ± 0.53	0.53 ± 0.07	155.33 ± 1.15	7.73 ± 0.08	3.01 ± 0.30	1.14 ± 0.03	0.86 ± 0.01	0.06 ± 0.01	0.01 ± 0.00	0.036 ± 0.00	0.022 ± 0.00
	5	11.01 ± 0.16	9.30 ± 0.73	1.00 ± 0.07	155.33 ± 1.15	7.69 ± 0.07	2.96 ± 0.21	1.17 ± 0.03	0.86 ± 0.00	0.06 ± 0.01	0.02 ± 0.00	0.034 ± 0.00	0.023 ± 0.00
	10	11.00 ± 0.16	9.03 ± 0.94	1.57 ± 0.06	156.33 ± 2.08	7.70 ± 0.05	2.71 ± 0.41	1.14 ± 0.00	0.86 ± 0.01	0.06 ± 0.01	0.02 ± 0.00	0.032 ± 0.00	0.024 ± 0.00
Two-way ANOVA	Month	***	***	NS	***	**	***	***	*	***	***	***	***
	Depth	NS	NS	*	NS	NS	NS	NS	NS	NS	NS	NS	NS
	Month × Depth	NS	***	NS	**	NS	NS	NS	*	NS	NS	NS	NS

T = water temperature; DO = dissolved oxygen; TN = total nitrogen; TP = total phosphorus. Data showed are means ± standard deviations (*n* = 3). * *p* < 0.05; ** *p* < 0.01; *** *p* < 0.001 represent statistical significance using two-way ANOVA. NS means no statistical significance.

**Table 2:** Three individual network properties of phytoplankton communities at the genus level.

Parameters	October	November	December
Average degree	0.824	0.778	0.875
Average weighted degree	0.805	0.758	0.843
Network diameter	3	2	3
Graph density	0.051	0.046	0.058
Modularity	0.73	0.796	0.597
Connected components	6	6	5
Average clustering coefficient	0.118	0.167	0.062
Average path length	1.435	1.222	1.5
Nodes	17	18	16
Edges	15	15	14

**Table 3:** Eukaryotic community diversity and richness estimators of Jinpen reservoir from October to December in 2018.

Month	Water Depth (m)	Reads Number	OTUs	0.97 Level			
				*Chao*1	Shannon Diversity (*H*’)	Simpson Diversity (D)	Coverage (%)
October	0	48,084	365	425 (398, 497)	2.92 (2.9, 2.94)	0.215 (0.211, 0.219)	99
	2	48,874	218	228 (221, 254)	2.38 (2.36, 2.4)	0.311 (0.306, 0.315)	99
	5	58,482	256	267 (259, 291)	3.26 (3.24, 3.28)	0.165 (0.163, 0.168)	99
	10	48,125	222	236 (225, 278)	3.15 (3.13, 3.17)	0.181 (0.178, 0.185)	99
November	0	36,313	132	143 (135, 176)	2.12 (2.1, 2.15)	0.381 (0.375, 0.387)	99
	2	44,037	304	308 (305, 322)	4.3 (4.29, 4.31)	0.026 (0.025, 0.026)	99
	5	31,597	415	476 (450, 523)	4.18 (4.16, 4.2)	0.034 (0.033, 0.035)	99
	10	47,074	378	406 (392, 435)	3.37 (3.35, 3.39)	0.151 (0.147, 0.154)	99
December	0	30,176	169	184 (174, 218)	3.8 (3.79, 3.82)	0.052 (0.050, 0.053)	99
	2	33,106	393	444 (421, 487)	4.17 (4.16, 4.19)	0.033 (0.032, 0.034)	99
	5	34,724	365	429 (401, 478)	3.58 (3.56, 3.6)	0.098 (0.096, 0.100)	99
	10	43,256	393	443 (421, 484)	3.98 (3.97, 4)	0.047 (0.046, 0.048)	99

**Table 4:** Network parameters of eukaryote community compositions based on the 18S DNA sequence at the genus level (R = 0.8).

Parameters	October	November	December
Average degree	2.194	2	3.138
Average weighted degree	2.115	1.928	2.99
Network diameter	14	6	12
Graph density	0.033	0.028	0.049
Modularity	0.743	0.789	0.584
Connected components	7	6	2
Average clustering coefficient	0.224	0.284	0.11
Average path length	3.777	2.228	4.881
Nodes	67	72	65
Edges	147	144	204

## Supplementary Materials

Supplementary materials can be found at https://www.mdpi.com/1660-4601/17/4/1128/s1. Figure S1: Images of phytoplankton under microscopy.
