# Supplementary material for: Community Compositions of Phytoplankton and Eukaryotes during the Mixing Periods of a Drinking Water Reservoir: Dynamics and Interactions
Source: Int J Environ Res Public Health. 2020 Feb 11;17(4):1128. doi: 10.3390/ijerph17041128 (PMC7068298; doi:10.3390/ijerph17041128)
Supplement: Supplementary file 1 [file ijerph-17-01128-s001.pdf]

Article

Community Compositions of Phytoplankton and Eukaryotes during the Mixing Periods of Drinking Water Reservoir: Dynamics and Interactions

Miaomiao Yan[1], Shengnan Chen[1,*], Tinglin Huang[1,*], Baoqin Li[2], Nan Li[1], Kaiwen Liu[1], Rongrong Zong[1], Yutian Miao[1], and Xin Huang[1]

[1] Shaanxi Key Laboratory of Environmental Engineering, Key Laboratory of Northwest Water Resource, Environment and Ecology, MOE, Xi'an University of Architecture and Technology, Xi'an 710055, Shaanxi Province, China; Ymmzsj@163.com (M.M.Y); kevin_wood1989@163.com (N.L); lkw088866@163.com (K.W.L); Rongrongzy_2019@163.com (R.R.Z); miaoyutian728@163.com (Y.T.M); xin_huang@xauat.edu.cn (X.H).

[2] Guangdong Key Laboratory of Integrated Agro-environmental Pollution Control and Management, Guangdong Institute of Eco-environmental Science and Technology, Guangzhou 510650, Guangdong Province, China; bqli@soil.gd.cn (B.Q.L)

*Author to whom correspondence should be addressed; E-Mail: chenshengnan@xauat.edu.cn (S.N.C.); huangtinglin@xauat.edu.cn (T.L.H); Tel.: +86-29-82202854 (S.N.C.); +86-29-82201038 (T.L.H.); Fax.: +86-29-82202729 (S.N.C & T.L.H.)

Supplementary information

Fig. S1

Supplementary information

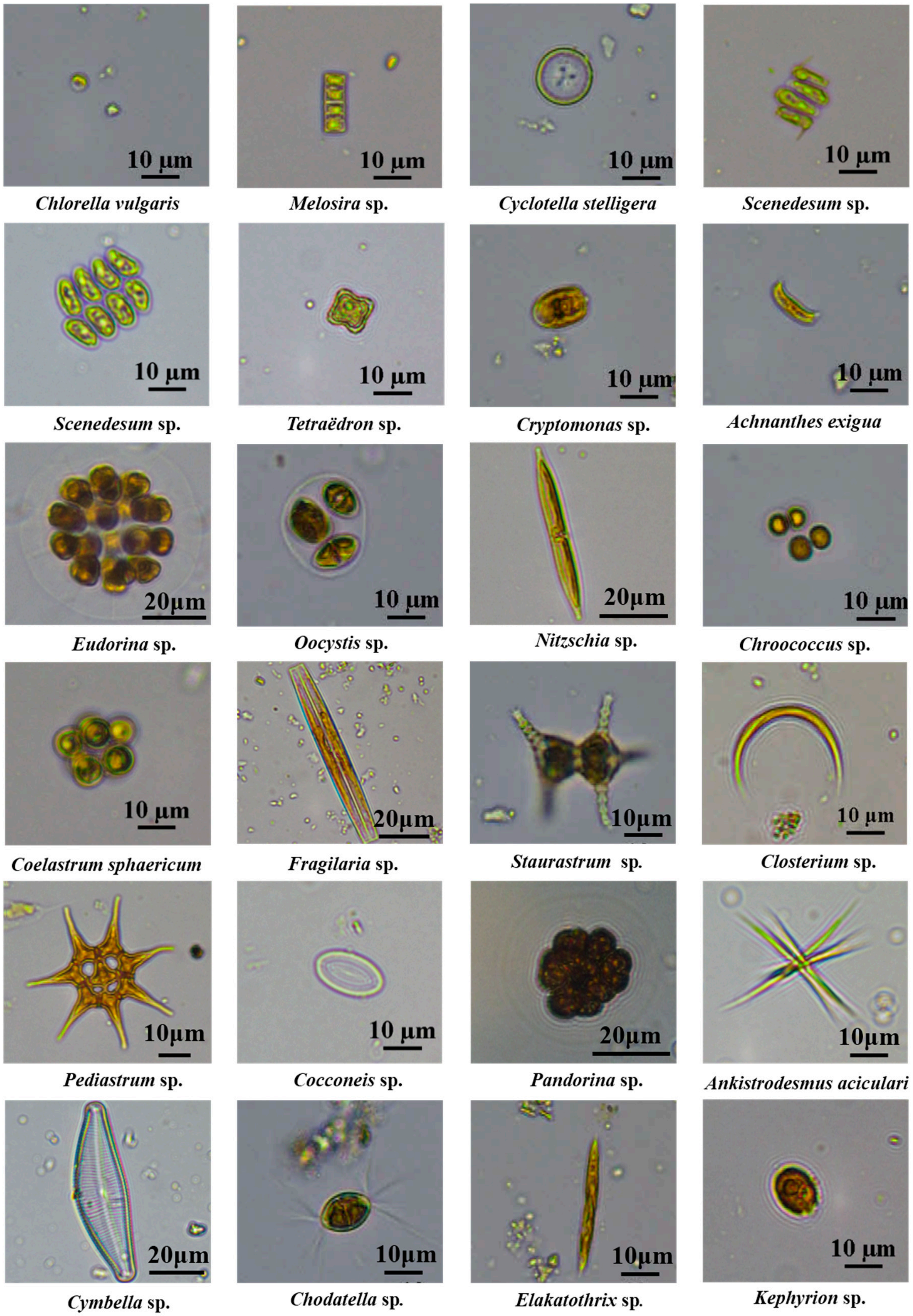

Figure S1 Images of phytoplankton under microscopy.
